# Strain-dependence of the Angelman Syndrome phenotypes in *Ube3a* maternal deficiency mice

**DOI:** 10.1038/s41598-017-08825-x

**Published:** 2017-08-16

**Authors:** Heather A. Born, An T. Dao, Amber T. Levine, Wai Ling Lee, Natasha M. Mehta, Shubhangi Mehra, Edwin J. Weeber, Anne E. Anderson

**Affiliations:** 1 Cain Foundation Laboratories, Jan and Dan Duncan Neurological Research Institute at Texas Children’s Hospital and Department of Pediatrics, Baylor College of Medicine, Houston, TX USA; 2 Department of Neuroscience, Baylor College of Medicine, Houston, TX USA; 3 Rice University, Houston, TX USA; 4 USF Health Byrd Alzheimer’s Institute, Department of Molecular Pharmacology and Physiology, University of South Florida, Tampa, FL USA; 5 Department of Neurology, Baylor College of Medicine, Houston, TX USA

## Abstract

Angelman syndrome (AS) is a genetic neurodevelopmental disorder, most commonly caused by deletion or mutation of the maternal allele of the *UBE3A* gene, with behavioral phenotypes and seizures as key features. Currently no treatment is available, and therapeutics are often ineffective in controlling AS-associated seizures. Previous publications using the *Ube3a* maternal deletion model have shown behavioral and seizure susceptibility phenotypes, however findings have been variable and merit characterization of electroencephalographic (EEG) activity. In this study, we extend previous studies comparing the effect of genetic background on the AS phenotype by investigating the behavioral profile, EEG activity, and seizure threshold. AS C57BL/6J mice displayed robust behavioral impairments, spontaneous EEG polyspikes, and increased cortical and hippocampal power primarily driven by delta and theta frequencies. AS 129 mice performed poorly on wire hang and contextual fear conditioning and exhibited a lower seizure threshold and altered spectral power. AS F1 hybrid mice (C57BL/6J × 129) showed milder behavioral impairments, infrequent EEG polyspikes, and fewer spectral power alterations. These findings indicate the effect of common genetic backgrounds on the *Ube3a* maternal deletion behavioral, EEG, and seizure threshold phenotypes. Our results will inform future studies on the optimal strain for evaluating therapeutics with different AS-like phenotypes.

## Introduction

Angelman Syndrome (AS) is a rare neurogenetic disorder that affects approximately 1 in 20,000 births with an equal incidence rate in genders (National Organization for Rare Disorders). The clinical diagnosis of AS includes developmental delay, intellectual disability, impaired motor function, dysmorphic facial features, and seizure phenotypes^1^. The main cause of AS is a genetic abnormality in the coding sequence of the ubiquitin protein ligase E3A (*UBE3A*) gene found on chromosome 15. Ube3a controls protein degradation toward the proteasomal pathway via the ubiquitin-tagging system. Due to genomic imprinting, the maternal allele of *UBE3A* is expressed and the paternal allele silenced. Defects in the imprinting center, deletion or mutation of the maternal *UBE3A*, or paternal uniparental disomy are known causes of the clinical phenotype of AS. The etiology is unknown in roughly 10% of AS patients^2^. The most common cause of AS, occurring in 70–80% of AS patients, is maternal deletion of region 15q11-q13, which encodes Ube3a protein^3, 4^. Epilepsy, which is often drug-resistant, is seen in about 80% of the AS population, and often co-occurs with chromosomal deletion^5, 6^. Additionally, distinct EEG patterns are reported in AS patients including intermittent rhythmic delta and theta waves as well as interictal epileptiform discharges^7, 8^.

Previous studies reported motor dysfunction, learning and memory impairment, and locomotor hypoactivity in mice with a maternally inherited *Ube3a* deletion in a variety of neurobehavioral tests^9–11^. Unlike the behavioral phenotypes, the seizure and EEG phenotypes have not been well-characterized in AS models and merit further study. Previously, spontaneous seizures were observed during handling and an increased susceptibility to audiogenic induced seizures was quantified in *Ube3a* maternally deficient AS mice by Jiang *et al*. in 1998. Another study reported spontaneous seizures in the related mouse model with a large deletion extending from *Ube3a* to *Gabrb3* ^12^, suggesting perhaps an exacerbation of the seizure phenotype owing to functional disruption of GABAergic neurotransmission in addition to the effects of *Ube3a* loss.

In order to effectively screen for novel therapeutics, a model that closely recapitulates the AS clinical phenotypes is needed. However, an issue related to AS mouse models is that the behavioral phenotype has been inconsistent in previously published studies^11, 13, 14^. The varying results found previously in AS mice suggest a need for in-depth characterization of the model to determine the optimal measures by which to evaluate AS biomarkers and potential rescue of these phenotypes with novel therapeutics. Although both models show almost no Ube3a protein expression in the brain of maternal-deficient mice, Miura *et al*.^13^ used an AS model with a mutation targeting different exons than the model used in this study, which was developed by Jiang *et al*.^9^, which should be taken into account when comparing previous studies characterizing behavioral and seizure phenotypes as the difference in mutation may affect phenotype expression. Furthermore, the background strain the model is bred on is known to affect behavior and audiogenic seizure phenotypes^9, 11^. Huang *et al*.^11^ performed comparative studies to determine the effect of C57BL/6J and 129 backgrounds on motor, sensorimotor gating, and learning and memory behaviors. We have built upon and extended previous work with EEG studies in addition to behavioral testing. Recent findings in a model with GABAergic *Ube3a* deficiency showed an increase in cortical EEG total and delta power similar to findings in AS humans^15^. However, there has not been a detailed evaluation of the EEG background rhythms, including hippocampal activity and different times in the circadian cycle, in the *Ube3a* maternal deficiency AS mouse model. In humans with AS, the intrinsic EEG background activity is typically disrupted often with additional epileptiform abnormalities. Thus, in these studies, we performed a comprehensive investigation of the behavioral, EEG, and seizure phenotypes in heterozygous mice with *Ube3a* maternal deficiency (AS mice) originally generated by Jiang *et. al*. in 1998 on three different backgrounds: C57BL/6 (B6), 129, and F1 hybrid (cross between C57BL/6 and 129). Interestingly, we found that there were pronounced strain-dependent differences in the AS phenotypes. Our findings are important for future studies in the context of screening novel therapeutics using this AS mouse model.

## Results

### AS mice have strain-dependent differences in activity

**Figure 1:**
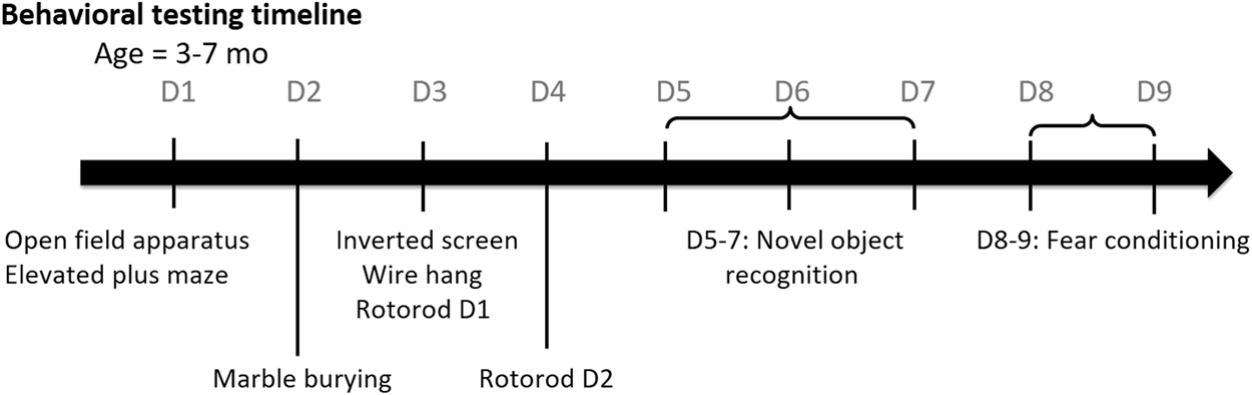
Timeline for behavioral battery of activity, anxiety, motor, and learning and memory tests.

**Figure 2:**
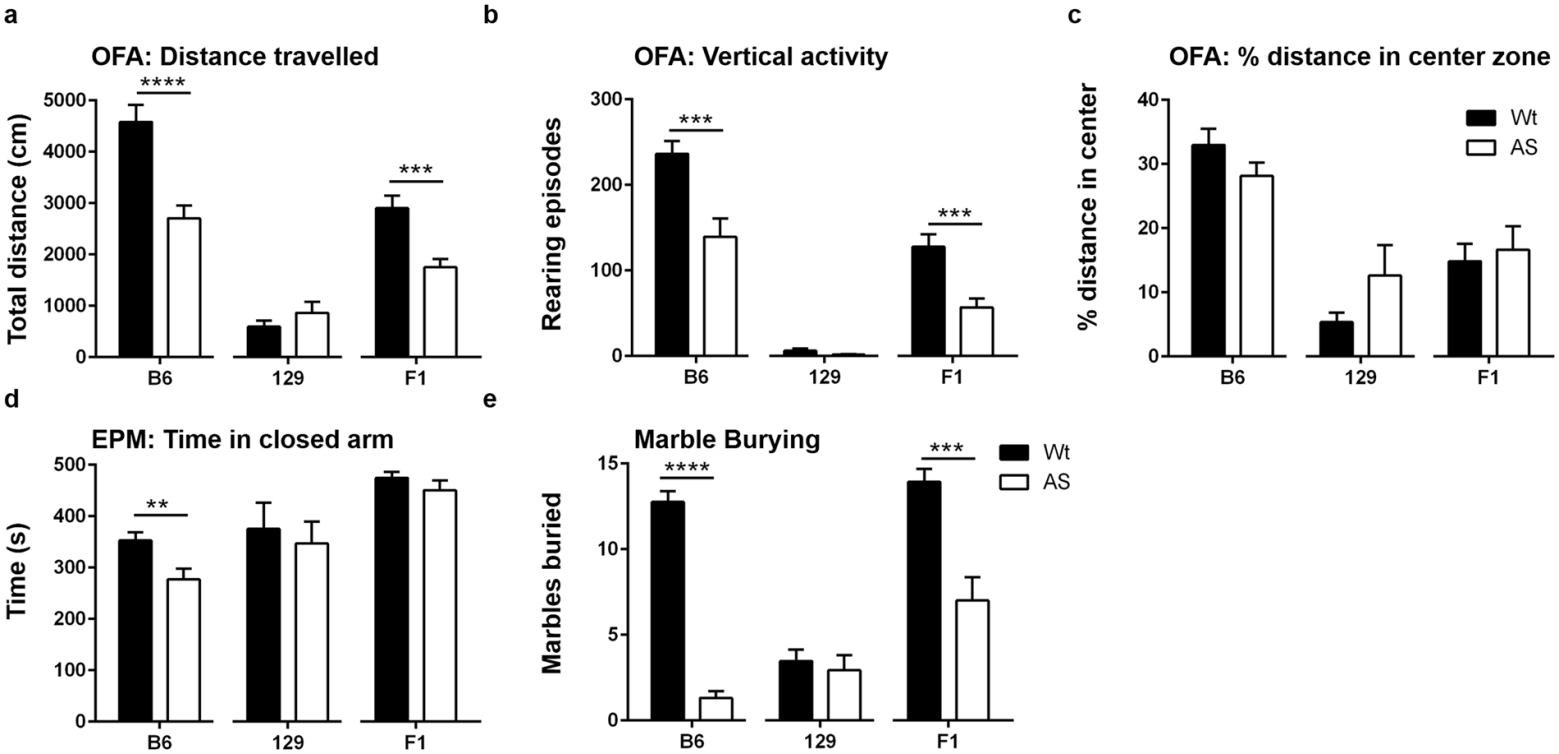
Strain dependent changes in activity, anxiety, and marble burying found in AS mice. Open field assessment (OFA), elevated plus maze (EPM), and marble burying tests highlighted differences between Wt and AS mice, as well as differences in activity levels between background strains. (**a,b**) Both B6 and F1 hybrid AS mice showed significant hypoactivity in the open field through distance travelled and number of rearing episodes (vertical activity). (**c**) No differences were found between Wt and AS mice in the OFA measure of anxiety, % distance travelled in center zone. (**d**) B6 AS mice spent significantly less time in the closed arms of the EPM. (**e**) B6 and F1 hybrid AS mice buried significantly less marbles than Wt mice. The 129 mice showed low amounts of locomotor activity and marble burying compared to the B6 and F1 hybrid mice. For each strain, AS and Wt mice were compared with a Student’s t-test and data are shown as the mean ± SEM, *p < 0.05, **p < 0.01, ***p < 0.001, ****p < 0.0001.

The behavioral battery used is described in Fig. 1. In the open field assay (OFA), AS mice of B6 and F1 backgrounds were hypoactive as indicated by reduced total distance travelled (Fig. 2a) (B6, Wt: 4580 ± 333.8 cm, AS: 2708 ± 247.7 cm, p < 0.0001; F1, Wt: 2901 ± 243.4 cm, AS: 1753 ± 156 cm, p = 0.005) and fewer rearing episodes (Fig. 2b) (B6, Wt: 236.3 ± 14.85, AS: 139.4 ± 21.23, p = 0.0008; F1, AS: Wt: 127.5 ± 14.80, 56.93 ± 10.39, p = 0.0005) compared to their Wt littermates. Both Wt and AS mice of 129 background travelled similar distances (Fig. 2a) (Wt: 592.2 ± 122.5 cm, AS: 866.5 ± 215.7 cm) and exhibited a similar number of rearing episodes (Fig. 2b) (Wt: 6.000 ± 2.724, AS: 1.733 ± 0.5561) in the OFA. Overall the 129 Wt and AS mice had much lower activity levels compared to B6 and F1 mice, and there was no significant difference on the 129 background.

### AS mice exhibit strain-dependent differences in anxiety-like behavior

We found no difference between genotypes on any backgrounds in the anxiety measure of % distance in center area of the OFA (Fig. 2c) (B6, Wt: 32.98 ± 2.536%, AS: 28.17 ± 2.07%; 129, Wt: 5.360 ± 1.442%, AS: 12.62 ± 4.756%; F1, Wt: 14.85 ± 2.719%, AS: 16.67 ± 3.612%). However, in the elevated plus maze (EPM), the B6 AS mice spent significantly less time in the closed arms compared to B6 Wt mice (Fig. 2d) (Wt: 352.5 ± 16.36 s, AS: 276.7 ± 20.85 s, p = 0.0077). This trend was not observed in the 129 and F1 backgrounds as these mice spent similar amounts of time in the closed arms regardless of genotype (129, Wt: 375.1 ± 51.22 s, AS: 347.0 ± 42.34 s; F1, Wt: 474.8 ± 11.36 s, AS: 450.6 ± 19.13 s).

### AS mice have strain-dependent differences in the marble burying assay

Marble burying is an assay for repetitive/perseverative digging behavior^16^. In our study, B6 and F1 AS mice buried significantly less marbles compared to Wt controls (Fig. 2e) (B6, Wt: 12.75 ± 0.6487, AS: 1.313 ± 0.4054, p < 0.0001, F1, Wt: 13.93 ± 0.7651, AS: 7.000 ± 1.373, p = 0.0001). This abnormality was not observed in the 129 background as both genotypes buried a comparable number of marbles (Wt: 3.467 ± 0.6822, AS: 2.933 ± 0.8916). Marble burying has been shown to not correlate with overall activity or other measures of anxiety, suggesting our results are not simply a reflection of the overall lower activity seen in the AS mice.

### AS mice have strain-dependent impairments on wire hang and accelerating rotarod

**Figure 3:**
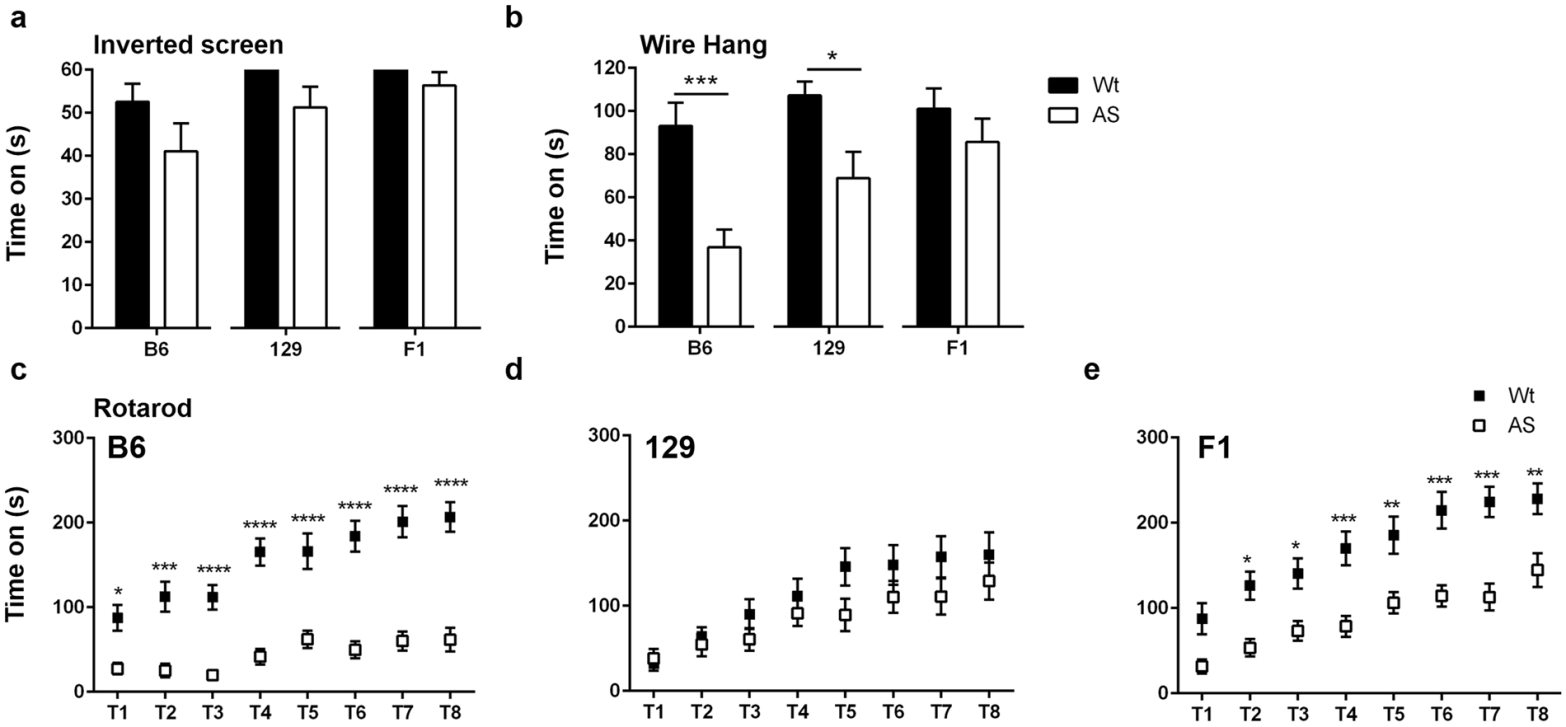
AS mice from all three backgrounds performed poorly on motor testing. (**a**) Adult AS mice from all three backgrounds performed normally on the inverted screen test. (**b**) AS mice from the B6 and 129 backgrounds spent significantly less time on the wire hang test before fall compared to Wt mice. For each strain, AS and Wt mice were compared with a Student’s t-test and data are shown as the mean ± SEM, *p < 0.05, ***p < 0.001. (**c–e**) When tested with the accelerating rotarod protocol, B6 and F1 hybrid mice performed significantly worse than Wt mice. Rotarod results were analyzed with 2-way ANOVA and Bonferroni posttest; n = 15–16 mice/group; *p < 0.05, **p < 0.01, ***p < 0.001, ****p < 0.0001.

For all backgrounds, we found no difference between genotypes in the latency to fall from the inverted screen (Fig. 3a) (B6, Wt: 52.58 ± 4.132, AS: 41.05 ± 6.478; 129, Wt: 60.00 ± 0.000, AS: 51.27 ± 4.805; F1, Wt: 60.00 ± 0.000, AS: 56.36 ± 3.058). However, we found that in the wire hang test, the latency to fall of B6 and 129 AS mice was significantly reduced compared to that of their Wt littermates (Fig. 3b) (B6, Wt: 93.14 ± 10.80 s, AS: 36.85 ± 8.179 s, p = 0.0002; 129, Wt: 107.2 ± 6.425 s, AS: 68.98 ± 12.21s, p = 0.0114). F1 Wt and AS mice had a similar latency to fall in the wire hang assay (Fig. 3b) (Wt: 101.1 ± 9.339 s, AS: 85.68 ± 10.79 s). On the accelerating rotarod, AS mice of B6 and F1 backgrounds displayed significantly impaired motor learning and memory as shown by a reduced latency to fall compared to strain-matched Wt controls (Fig. 3c,e). At the age tested, there was no significant difference between Wt and AS mice on the 129 background. Motor learning and memory was impaired in AS mice, especially in the B6 AS mice. For example, during trial 8, the B6 AS mice latency to fall was an average of 61.50 s while the average latency to fall for B6 Wt mice was 206.6s (Fig. 3c). Motor impairments were most obvious in the B6 background, least obvious in the 129 background, and intermediate in the F1 background.

### AS mice have strain-dependent impairments in novel object recognition and fear conditioning

**Figure 4:**
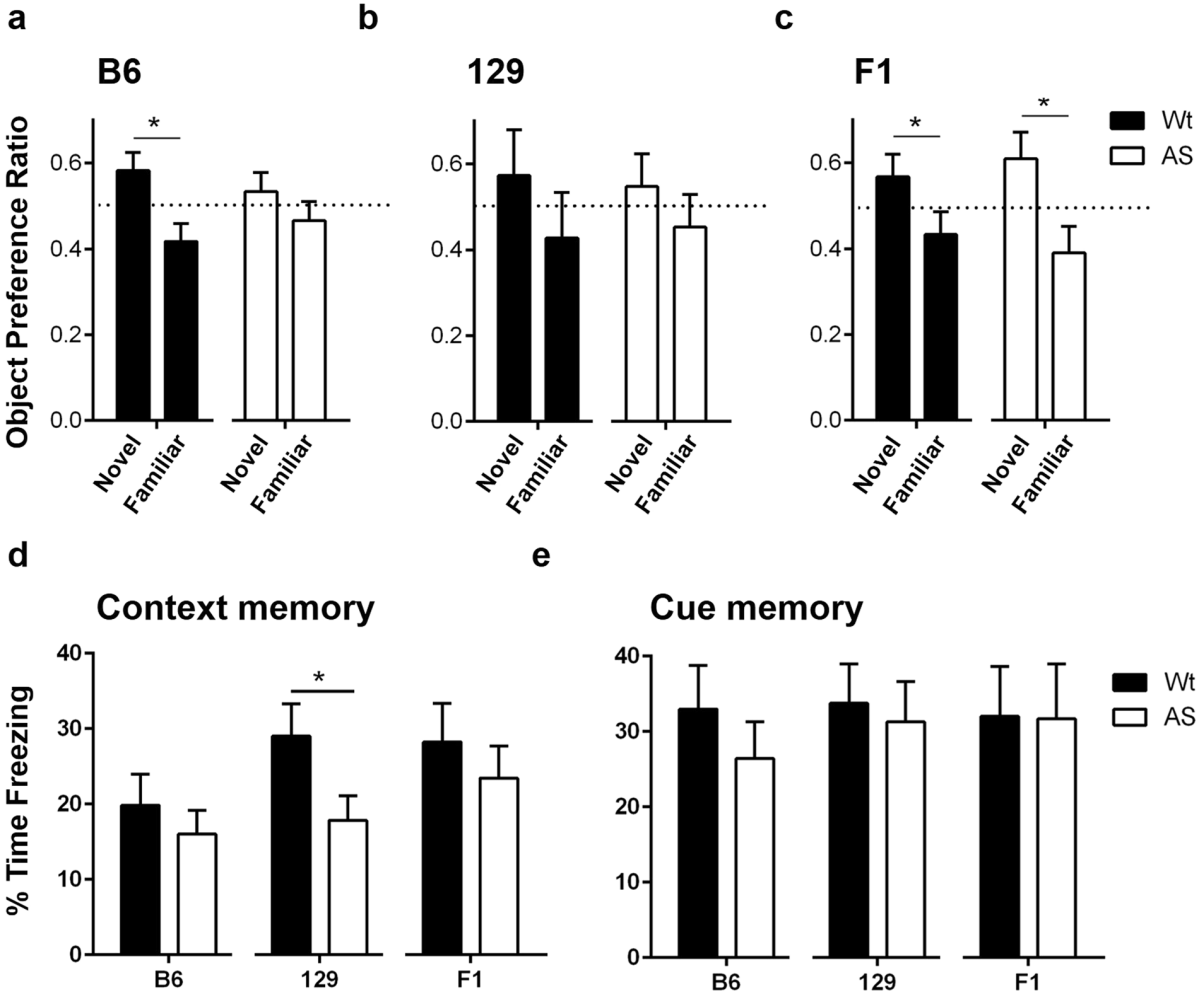
AS mice showed strain-dependent learning and memory deficits. (**a**) The B6 Wt mice spent significantly more time with the novel object and were successful in the NOR task, while the B6 AS mice showed a similar preference for both objects. (**b**) Both the 129 AS and Wt mice performed poorly on NOR. (**c**) The F1 hybrid AS and Wt mice both showed a significant preference for the novel object. (**d**) The 129 AS mice showed a significant decrease in freezing behavior compared to Wt mice during the 24 hour context test indicating decreased fear memory. No significant difference in % time freezing was seen during the fear conditioning context test for the B6 and F1 hybrid mice. (**e**) Mice from all three backgrounds showed no difference in freezing behavior during cue testing 26 hours after fear conditioning training. For each strain, AS and Wt mice were compared with a Student’s t-test and data are shown as the mean ± SEM; n = 15–16 mice/group; *p < 0.05.

In the novel object recognition (NOR) test, B6 AS mice showed no preference toward the novel object, while B6 Wt mice showed a significant preference for the novel object during testing (Fig. 4a) (p = 0.0093). Both Wt and AS 129 mice failed to show a preference for the novel object; perhaps due to their innate locomotor hypoactivity (Fig. 4b). In contrast with the B6 background, both genotypes on F1 background showed a significant preference for the novel object (Fig. 4c) (Wt: p = 0.0253, AS: p = 0.017).

In the fear conditioning (FC) task, only AS mice on the 129 background showed a significant impairment in long-term contextual memory and froze less than Wt mice (Wt: 29.03 ± 4.250%, AS: 17.84 ± 3.280%, p = 0.0465) (Fig. 4d). Cue memory was unaffected as both genotypes on all backgrounds froze the same amount of time during cue testing (Fig. 4e). No significant differences were found in baseline freezing prior to footshock during training (B6, Wt: 0.00 ± 0.00, AS: 0.626 ± 0.290; 129, Wt: 0.00 ± 0.00, AS: 0.74 ± 0.74; F1, Wt: 0.149 ± 0.149, AS: 0.460 ± 0.460).

### AS mice have strain-dependent epileptiform EEG findings in AS mice

**Figure 5:**
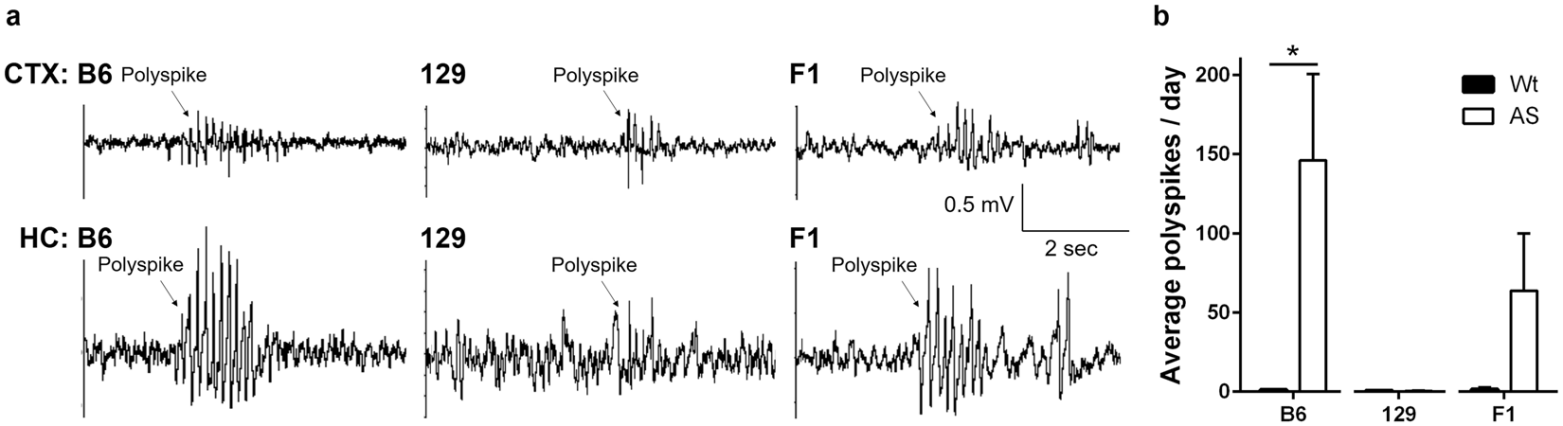
Analysis of spontaneous epileptiform activity. (**a**) Representative traces of spontaneous polyspike burst activity were identified in AS mice of all three background strains. The B6 and F1 hybrid AS mice typically exhibited more pronounced and frequent polyspike events compared with 129 AS mice. The Wt mice of all backgrounds did not show abnormal EEG activity. (**b**) Quantification of polyspike activity in all three strains during continuous vEEG recording (24 hrs for 5 days). Both B6 and F1 AS mice exhibited more polyspike events in cortical and hippocampal EEG traces compared with 129 AS mice. The B6 AS mice showed the most pronounced and frequent polyspikes. For each strain, AS and Wt mice were compared with a Student’s t-test with Welch’s correction where variances were significantly different and data are shown as the mean ± SEM; n = 3–7 mice/group; *p < 0.05. CTX: cortex, HC: hippocampus.

We quantified spontaneous epileptiform activity over the 5–7 day video synchronized EEG (vEEG) continuous monitoring period by counting polyspikes in cortical and hippocampal EEG activity from Wt and AS mice on all backgrounds (Fig. 5a). Wt mice exhibit normal EEG activity, while AS mice showed spontaneous polyspikes. This polyspike activity was most frequent in the B6 background with an average of 146 ± 54.64 events/day in B6 AS mice compared to 1.04 ± 0.20 events/day in B6 Wt mice (p = 0.0453), while fewer of these abnormal events were observed in the F1 hybrid (Wt: 1.53 ± 1.13 events/day compared to AS: 63.77 ± 36.18 events/day; p = 0.1461). The 129 AS mice observed during continuous long-term vEEG recordings exhibited infrequent abnormal polyspike activity (Wt: 0.60 ± 0.23 events/day compared with AS: 045 ± 0.22 events/day; p = 0.6638).

### AS mice have strain-dependent responses to convulsant stimulation

**Figure 6:**
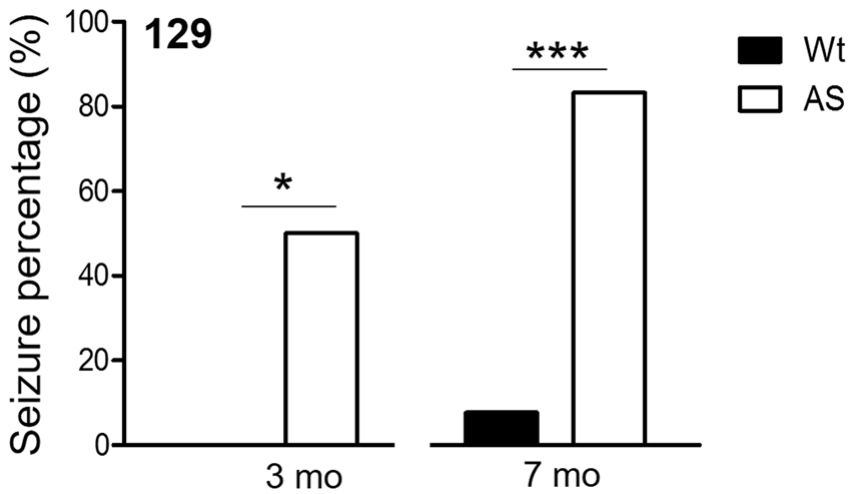
129 AS mice were vulnerable to audiogenic seizures and exhibited wild running and tonic-clonic seizures when exposed to 140 dB sound in an enclosed chamber. The AGS phenotype affected a greater percentage of mice in the 7 mo old age group when compared with younger 3 mo old mice (n = 11–13 / group). Fisher’s test was used for analysis of seizure percentage at each designated age; n = 11–12 mice/group; *p < 0.05, ***p < 0.001.

Since we observed no spontaneous seizures in AS mice on all backgrounds over 5–7 days of continuous 24 hr monitoring and screening for spontaneous seizure activity, we induced audiogenic seizures (AGS) using a 140 dB emitting alarm in both Wt and AS mice. We observed a strain-specific robust AGS phenotype. Young AS 129 mice (3–3.5 mo) developed AGS, which were not seen in AS B6 or F1 mice (Fig. 6, data not shown for B6 and F1 mice) (n = 11–13 / group). Audiogenic seizures in AS mice have been previously reported^9^. In our study, 50% of 3–3.5 mo old 129 AS mice developed robust AGS seizures with wild running or tonic-clonic behavior (p = 0.0137) while no Wt mice on any background, or B6 and F1 AS mice, exhibited seizure behaviors. Additionally, the percentage of AS 129 mice vulnerable to AGS induction increased to 83% in older (6.5–7 mo) mice (p = 0.0002) (Fig. 6).

**Figure 7:**
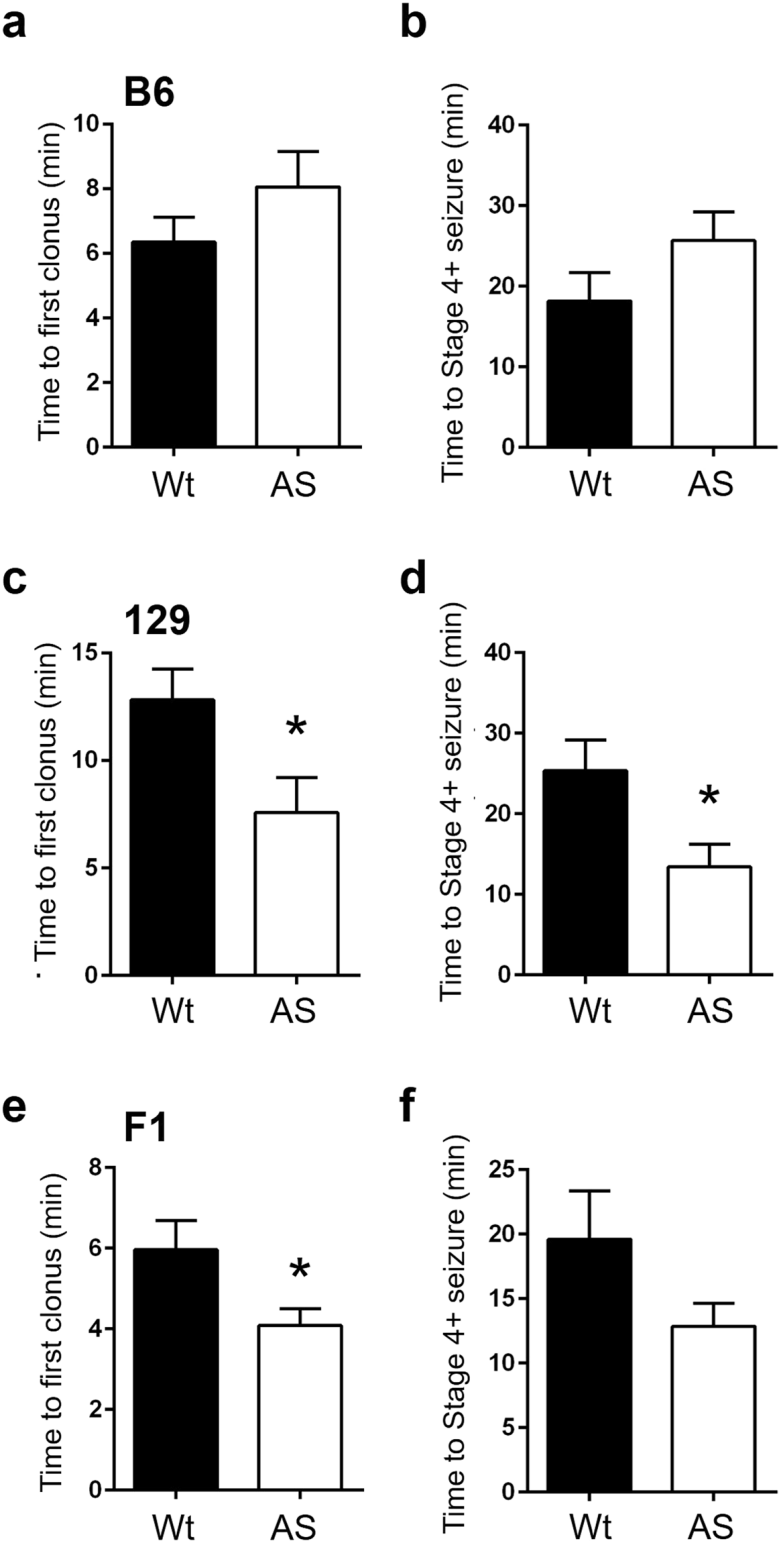
Seizure induction with KA. (**a,b**) KA induction (25 mg/kg) in Wt and AS mice in the B6 background (n = 12–13 / group). No significant difference was found between B6 Wt and AS mice for time to reach the first clonus (tail/head/limbs) (**a**) or time to reach stage 4 or greater seizure (**b**). (**c,d**) KA induction (40 mg/kg) in Wt and AS mice in the 129 background (n = 11–12 / group). 129 AS mice exhibited a significantly lower latency to reach the first visible clonus of tail/head/limb (**c**) and stage 4 or greater seizure (**d**) compared to Wt littermates. (**e,f**) KA induction (35 mg/kg) in Wt and AS mice in the F1 background (n = 11–12 / group). F1 AS mice exhibited a significantly lower latency to reach the first visible clonus of tail/head/limb (**e**) compared to their Wt littermates. No significant difference was found between F1 Wt and AS mice for latency to stage 4 or greater seizure (**f**). Data are presented as the mean ± SEM; Student’s t-test was used to compare between AS and Wt mice; n = 11–13 mice/group; *p < 0.05.

To test whether AS mice were more susceptible to a chemoconvulsant, we induced seizures in Wt and AS mice on all backgrounds. Kainate (KA) at 25 mg/kg, 40 mg/kg, and 35 mg/kg was used to induce seizures in the B6, 129, and F1 (n = 11–13 / group) backgrounds, respectively. The kainate dosage was calculated based on dose-response studies (data not shown). We observed a reduced time to first visible clonus following KA induction in 129 AS mice compared to their Wt controls (Wt: 12.82 ± 1.431 min compared with AS: 7.565 ± 1.643 min, p = 0.0246) (Fig. 7c). The decreased time to behavioral seizure in 129 AS mice persisted when comparing the latency to a stage 4 or greater seizure (Wt: 25.36 ± 3.783 min compared with AS: 13.40 ± 2.864 min, p = 0.0214) (Fig. 7d). We also observed a reduced time to first visible clonus following KA induction in F1 AS mice compared to their Wt controls (Wt: 5.965 ± 0.719 min compared with AS: 4.085 ± 0.417 min, p = 0.0385) (Fig. 7e). Outside of the time to first clonus in F1 AS mice, both Wt and AS mice of B6 and F1 backgrounds displayed similar latencies to seizure stages between genotypes when challenged with kainate (Fig. 7a,b,f)

### AS mice have strain-dependent quantitative differences in EEG power spectrum analysis

**Figure 8:**
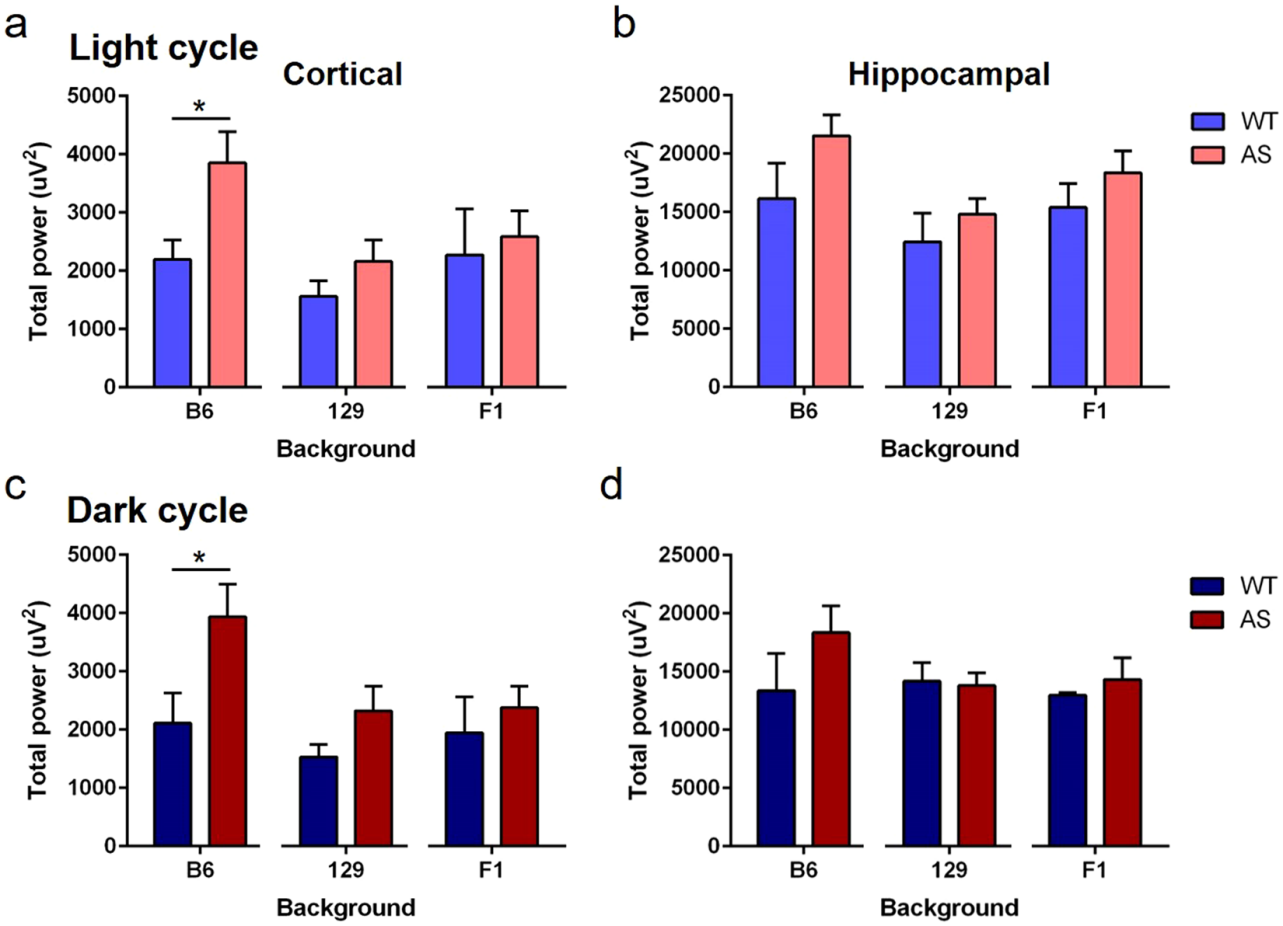
Total EEG spectral power in the cortex and hippocampus of Wt and AS mice on B6, 129, or F1 hybrid backgrounds. (**a**,**c**) Total EEG spectral power in cortex during the light (**a**) and dark (**c**) cycle was significantly increased in B6 AS mice compared to Wt controls (n = 9–11/group). No differences in total EEG spectral power between genotypes were observed in the recordings from cortex with 129 (n = 8–10/group) and F1 hybrid (n = 3–6/group) backgrounds. (**b,d**) Total EEG spectral power in hippocampus during the light (**b**) and dark (**d**) cycle was not significantly different in AS mice compared to the Wt controls for any strain. For each strain and time of day, Student’s t-test was used for analysis and data are presented as the mean ± SEM; *p < 0.5.

Upon visual inspection, qualitative differences were observed in baseline EEG activity between Wt and AS mice (Figs S1 and S2). Using EEG spectral quantification, we found an increase in total power of B6 AS mice in the cortex during both light and dark cycle (Fig. 8a and c) (Light cycle: Wt: 2192 ± 336.1 µV^2^ compared with AS: 3853 ± 537.9 µV^2^, p = 0.0193; Dark cycle: WT: 2111 ± 516.6 µV^2^ compared with AS: 3937 ± 561.9 µV^2^, p = 0.0296). No quantitative differences in total hippocampal power for either time of day were found between B6 AS and Wt mice (Fig. 8b and d), nor were differences between AS and Wt mice evident in cortical or hippocampal total power during the light or dark cycle from 129 and F1 mice (Fig. 8). These data suggest that *Ube3a* maternal deletion results in strain-dependent differences in EEG activity.

**Figure 9:**
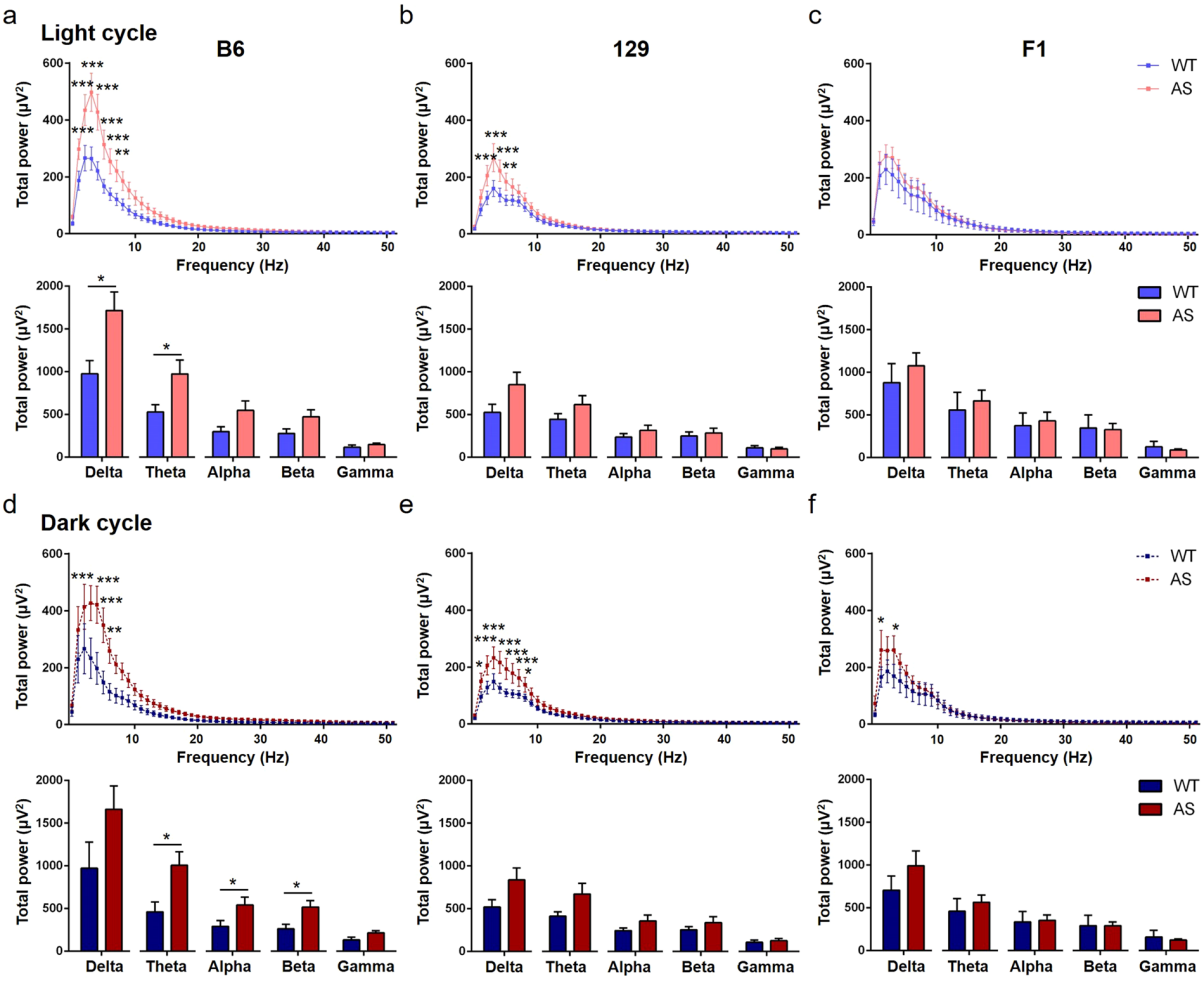
Detailed analysis of EEG spectral cortical power during a representative hour of the light cycle (**a–c**) and dark cycle (**d**,**e,f**) from AS mice compared to Wt mice. (**a,d**) There was an upward shift of the B6 AS spectral cortical power compared to the B6 Wt mice during both dark and light cycle. This shift was primarily driven by increased power in the delta and theta frequency range (n = 9–11/group). During the dark cycle (**d**), significant increases were also seen in the summed alpha and beta spectral bands in B6 AS mice. (**b,e**) An upward shift in power was also seen in 129 AS compared to 129 Wt mice, driven by increased power in the 1–7 Hz range, although no significant differences were seen in the summed frequency bands (n = 8–10/group). (**c,f**) No significant changes were seen between F1 AS and Wt mice during the light cycle, however increased power was seen in the delta frequency range in F1 AS mice during the dark cycle when compared to F1 Wt mice (n = 3–6 / group). 2-way ANOVA was used to compare the individual frequencies across the spectrum. Student’s t-test was used to compare between individual frequency bands and data are presented as the mean ± SEM; *p < 0.05, **p < 0.01, ***p < 0.001.

**Figure 10:**
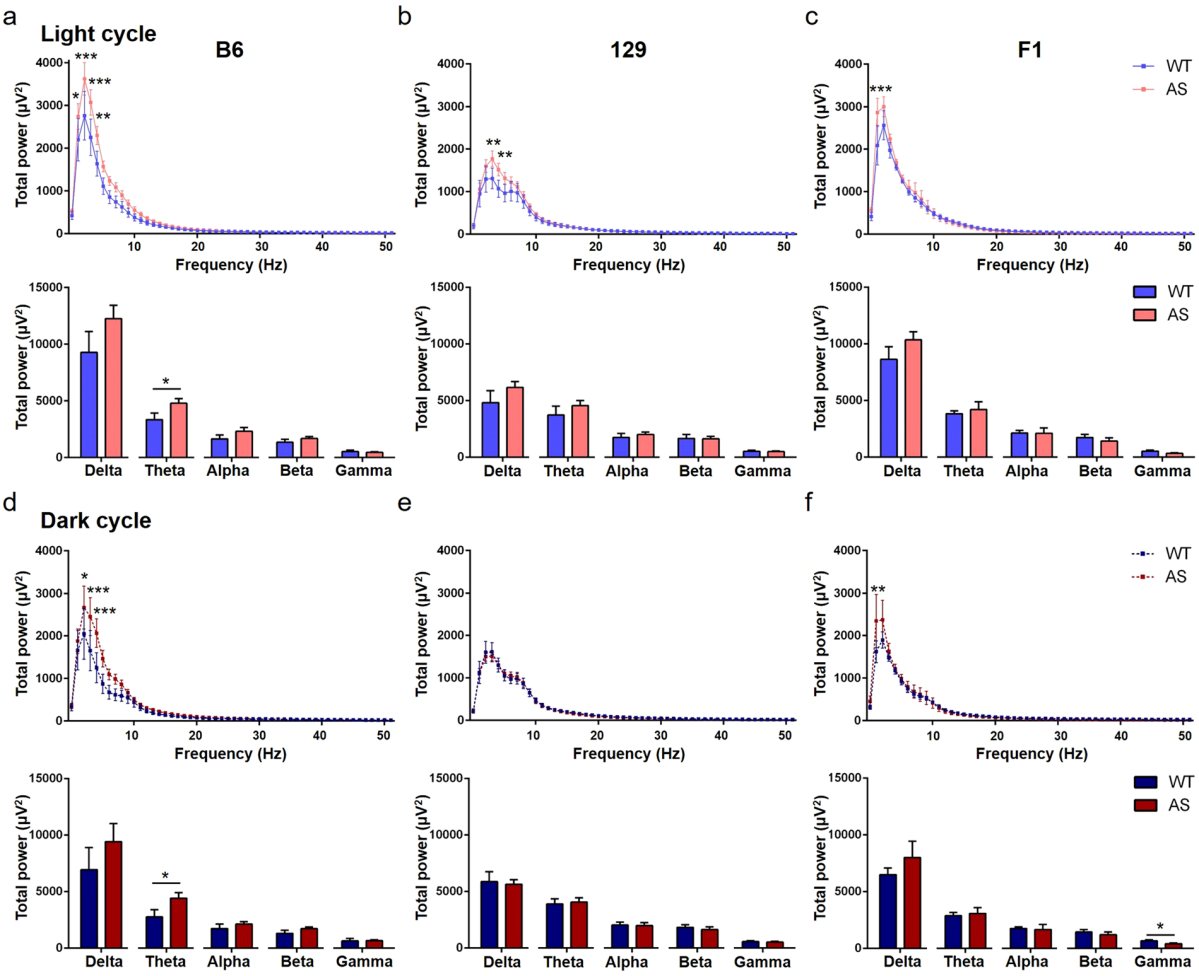
Detailed analysis of EEG spectral hippocampal power during a representative hour of the light cycle (**a–c**) and dark cycle (**d–f**) from AS mice compared to Wt mice. (**a,d**) There was an upward shift of the B6 AS mice spectral hippocampal power compared to the B6 Wt mice during both dark and light cycle. Increased power was seen in individual frequencies in the delta range and the summed theta bands (n = 9–11/group). (**b,e**) An upward shift in power was also seen in 129 AS compared to 129 Wt mice during the light cycle (B), driven by increased power in individual frequencies in the delta range, although no significant differences were seen in the summed frequency bands (n = 8–10/group). Hippocampal EEG activity during the dark cycle (**e**) was similar for 129 Wt and AS mice. (**c,f**) A significant increase in power at 1 Hz was seen during both light (**c**) and dark (**f**) cycles, while a significant decrease in the summed gamma spectral band was found in F1 AS mice only during the dark cycle (**f**) (n = 3–6 / group). 2-way ANOVA was used to compare the individual frequencies across the spectrum. Student’s t-test was used to compare between individual frequency bands and data are presented as the mean ± SEM; *p < 0.05, **p < 0.01, ***p < 0.001.

We extended our studies of total EEG power by examining the relative power of different frequencies in the cortex and the hippocampus (Figs 9 and 10). When comparing the Wt and AS mice during the light or dark cycle, B6 and 129 AS mice showed significant increases in cortical power due to genotype (p < 0.0001) and post-hoc differences at both times of day for multiple frequencies within the delta and theta range (Fig. 9a; Light cycle: B6, p < 0.001 at 1–6 Hz, p < 0.01 at 7 Hz; Fig. 9b; 129, p < 0.001 at 2–4 Hz, p < 0.01 at 5 Hz; Fig. 9d; Dark cycle: B6, p < 0.001 at 2–5 Hz, p < 0.01 at 6 Hz; Fig. 9e; 129, p < 0.001 at 2–6 Hz, p < 0.05 at 1 and 7 Hz). On the F1 background, no differences were found in the light cycle (Fig. 9c) and there was only a significant increase in power due to genotype (p = 0.0279) during the dark cycle (Fig. 9f; p < 0.05 at 1 and 3 Hz). The B6 AS mice differed in the cortical EEG power spectrum compared to Wt mice during the light cycle with significant increases in delta and theta power (Fig. 9a; Delta, Wt: 973.5 ± 154.2 µV^2^ compared with AS: 1715 ± 214.5 µV^2^, p = 0.0126; Theta, Wt: 527.5 ± 84.46 µV^2^ compared with AS: 973.2 ± 163.4 µV^2^, p = 0.0286). Additional increased power was seen in B6 AS mice in the alpha and beta frequencies during the dark cycle (Fig. 9d; Theta, Wt: 459.3 ± 116.8 µV^2^ compared with AS: 1005 ± 159.6 µV^2^, p = 0.015; Alpha, Wt: 289.5 ± 67.96 µV^2^ compared with AS: 540.2 ± 92.59 µV^2^, p = 0.0471; Beta, Wt: 261.1 ± 52.35 µV^2^ compared with AS: 516.6 ± 75.34 µV^2^, p = 0.0144). No significant differences were found in the summed spectral frequency bands for either light or dark cycle on the 129 and F1 backgrounds.

The hippocampus is involved in learning and memory, so we further evaluated hippocampal EEG frequency power spectrum. The B6 AS mice differed in the hippocampal EEG power spectrum with increased power in the delta frequency range compared to Wt mice during both the light and dark cycles (Fig. 10a; Light cycle: B6, genotype: p < 0.0001, p < 0.001 at 2 and 3 Hz, p < 0.01 at 4 Hz, p < 0.05 at 1 Hz; Fig. 10d; Dark cycle: B6, genotype: p = 0.0002, p < 0.001 at 3 and 4 Hz, p < 0.05 at 2 Hz). When comparing the summed spectral bands, increased power was seen in the theta band (Fig. 10a; Light cycle, Wt: 3338 ± 591.9 µV^2^ compared with AS: 4797 ± 409.5 µV^2^, p = 0.05; Fig. 10d; Dark cycle, Wt: 2755 ± 637.7 µV^2^ compared with AS: 4410 ± 503.6 µV^2^, p = 0.05). On the 129 background, significantly increased power was found in AS mice compared to Wt mice only during the light cycle (Fig. 10b; genotype: p = 0.0031, p < 0.01 at 3 and 4 Hz), while no differences were seen between 129 Wt and AS mice during the dark cycle (Fig. 10e) and no differences were found when comparing the summed spectral frequency bands. In line with our cortical dark cycle findings, posthoc analysis identified increased power for F1 AS compared to Wt mice in the delta range for both time points (Fig. 10c; Light cycle: F1, p < 0.001 at 1 Hz; Fig. 10f, Dark cycle: F1, p < 0.01 at 1 Hz), however when summed spectral bands were compared, the only difference was significantly decreased gamma power in F1 AS mice during the dark cycle (Fig. 10f; Dark cycle: F1, Wt: 654.8 ± 90.51 µV^2^ compared with AS: 406.6 ± 56.23 µV^2^, p = 0.0476). The significantly higher delta and theta power found in AS mice is similar to that reported in qualitative EEG assessments from humans with AS^17–19^.

## Discussion

Genetic mouse models of AS recapitulate both abnormal behavioral phenotypes and epileptiform activity, and are important for testing potential therapies as well as understanding the molecular mechanisms underlying AS phenotypes for directing therapeutic discovery. However, as previously reported, the AS phenotype is strain-dependent^9, 11^. In contrast with a previous study comparing the effect of strain background on AS-related behavioral phenotypes from Huang *et al*.^11^, adult mice from all three strains were subjected to an identical behavioral battery, although we did not evaluate performance on the same task at multiple ages. In our study, we demonstrated the effect of mouse strain background (B6, 129, and F1 B6 × 129 hybrid) on behavioral assays, spontaneous epileptiform activity, background EEG spectral power analysis, and provoked seizures in Wt and AS mice.

One finding that persists across all three background strains used in our study (B6, 129, and F1 hybrid) is increased weight compared to Wt mice of the same background (Fig. S3). Increased body weight is a consistent finding in adult AS mice^11, 14^ and has been reported in some adolescent and adult humans with AS^20–22^.

In contrast with the consistent finding of increased weight, we found strain-dependent differences in age- and test battery-matched behavioral assays. B6 and F1 AS mice were hypoactive and exhibited low marble-burying compared to Wt mice on B6 and F1 backgrounds, while Wt and AS 129 mice exhibited very low levels of activity and buried few marbles. While there were strain-specific differences in which test revealed impaired motor ability, AS mice from all three background strains did poorly on one or multiple motor tasks (inverted screen, wire hang, rotarod). In agreement with our findings, studies from other labs using B6 AS mice also have shown decreased locomotor activity in terms of both distance traveled and vertical movements, poor wire hang and rotarod performance, and decreased marble burying^11, 13, 14^. In our study, decreased anxiety-related behavior was seen in the EPM test with B6 AS mice. This is in agreement with decreased anxiety behavior in the OFA test shown previously by Huang *et al*., although there were different results in the time in center region during OFA testing with two behavioral cohorts separated by a week in age, suggesting decreased anxiety as measured by OFA testing may not be a very robust phenotype^11^.

Decreased locomotor activity in 129 mice compared to B6 mice has been reported previously by Huang *et al*. and is confirmed by our study^11^. Normal performance on OFA and EPM with impairments in rotarod and contextual fear conditioning have been previously found in the 129 AS mice^23^. However, rotarod performance comparable to Wt 129 mice has been previously reported, which is confirmed by our findings^11^. Differences in 129 AS rotarod assessment may be due to variations in training protocols (e.g. total number of trials or trials/day), and motor deficits in AS mice are likely age-dependent, as Huang *et al*.^11^ showed an age-dependent decrease in motor performance in B6 AS mice, although 129 AS mice were not tested with different cohorts at multiple ages. In line with our behavioral findings of motor deficits in F1 AS mice, impaired bar-crossing and rotarod performance in F1 AS hybrids has been reported previously^9^. Significant differences in gait when measured by footprint analysis has also been reported with F1 AS mice^9^. Prominent hallmarks of AS are motor-related developmental delay, jerky movements, and an ataxic gait^18, 20, 24^. In addition, while hyperactivity and restlessness can be problematic in younger children, older individuals with AS have been found to be hypoactive^20^.

To assess learning and memory performance, we used the NOR and FC tasks. The Morris water maze (MWM) test has frequently been used in AS model studies to investigate spatial learning and memory impairments. Numerous studies report poor MWM performance in both B6 and 129 AS mice, including an age-dependent deficit in MWM acquisition in B6 AS mice and normal performance in MWM acquisition followed by deficits in reversal MWM learning in 129 AS mice^11, 13, 23^. However, the NOR task evaluated here can be completed while mice are connected to EEG recording equipment and used to correlate behavioral performance and EEG activity within mouse, which prompted us to use the NOR task in our studies as an assessment of learning and memory.

The results are mixed when assessing AS mice with the contextual FC test. Decreased freezing has been reported for B6 AS mice in the context test^13, 14^, although normal freezing during the context test and poor performance on the cue test only also has been reported^11^. Impaired contextual FC has been previously found in 129 AS mice and F1 AS mice^9, 23^. In our study, we found no differences in FC for either context or cue tests with B6 AS or F1 AS mice, but did see significantly impaired contextual FC in 129 AS mice. Differences in reports of impaired FC may be due in part to testing methods and analysis.

In our study, there were no spontaneous seizures detected by continuous long term v-EEG monitoring in AS mice on any background strain, which contrasts with previous reports of seizures, abnormal spike wave activity, and slow wave discharges in AS mice and the high percentage of individuals with AS that have epilepsy^9, 24^. We identified abnormal epileptiform activity characterized by polyspike events in AS mice across all three backgrounds with a significantly increased frequency of polyspike events in B6 AS mice compared with Wt mice. To our knowledge, we are the first group to characterize and quantify this type of spontaneous epileptiform activity from electrodes placed over somatosensory cortex and depth electrodes in hippocampus for continuous long-term vEEG recordings in the *Ube3a* maternal deletion AS model. While not the same type of events, Mandel-Brehm and colleagues reported that juvenile AS B6 mice with a null mutation of *Ube3a* displayed large amplitude spiking events in the cortex^25^, and Miura *et al*. reported intermittent bursts of 4–5 Hz spike-wave discharges that were 5–12s in duration in hippocampal EEG of awake B6 AS mice^13^. Differences seen in EEG abnormalities may be due to differences between models and exons targeted for disruption in *Ube3a*. Interestingly, the B6 and F1 AS mice demonstrated significantly greater abnormal epileptiform polyspike activity compared with 129 AS mice, but there was no increased susceptibility to AGS in the B6 and F1 AS mice (data not shown).

There was increased AGS vulnerability only in 129 AS mice which is similar to that reported by Jiang *et al*.^9^. Mandel-Brehn *et al*. (2015), reported that 129 AS mice had severe AGS-induced seizures and death. They also found a lengthier recovery time from audiogenic-induced freezing behavior, a measure not used in our study, in AS juvenile mice on both B6 and F1 backgrounds^25^. These discrepancies in induced seizures and epileptiform activity are likely due to differences in the AGS induction protocol and analysis as well as the effects of hybrid background strains. The exact underlying differences that lead to different AS-like phenotypic expression in mouse strains are unclear. The separation of the vulnerability to induced seizures seen only in 129 AS mice from the abnormal spontaneous EEG findings seen in B6 and F1 mice may be due to disruptions in different relevant neural circuits. Future studies investigating differences in genetic modifiers and signaling cascades in AS mice from multiple backgrounds are needed to fully understand how different genetic make-up can affect neural circuitry and AS-like phenotypic expression.

Abnormal EEG findings are a hallmark feature in humans with AS. These abnormalities include an excess of delta and theta frequencies, which are quantifiable abnormalities using spectral analysis. We used long-term v-EEG to evaluate baseline electrographic activity and upon visual inspection of the EEG activity found strain-specific abnormal baseline EEG activity. In order to quantify these differences we used spectral power analysis. We assessed EEG activity during both the light cycle, when mice spend more time sleeping, and dark cycle, when mice are more active, to evaluate the effect of time of day on EEG spectral power. While the time of day, and state of the animal as an extrapolation from this, affected spectral power, many of the differences found in AS mice compared with Wt mice from the same background were similar in both the light and dark cycles. Much of the increased power seen in AS mice was due to changes in the delta and theta frequency range, although the specific frequencies varied slightly between light and dark cycles. Some exceptions to this include significantly increased cortical alpha and beta, but not delta, frequency bands specifically during the dark cycle in B6 AS mice, although there were trends towards increased power in these bands during the light cycle in B6 AS mice. Other examples of time-dependent differences include comparable 129 Wt and AS hippocampal EEG during the dark cycle while power at 3 and 4 Hz was significantly increased during the light cycle in 129 AS mice, and decreased hippocampal gamma activity in F1 AS compared to F1 Wt mice specifically during dark cycle. Overall, the spectral analysis revealed an increase in total cortical power in B6 AS compared to B6 Wt mice. Our findings support and strengthen previous reports from local field potential recordings in the V1 layer 4 showing a trend towards increased total power in the *Ube3a* ^*STOP/p*+^ (suppressed neuronal *Ube3a*) and *Ube3a* ^*FLOX/p*+^ *::Gad2-Cre* (GABAergic specific *Ube3a* deletion) on the 129S2/SvPasCrl background compared to control mice^15^. Similar to our behavioral assessments, the B6 AS mice exhibited the most severe abnormalities in intrinsic EEG background rhythms while, to a lesser degree, the 129 and F1 AS mice displayed increased power in the delta and theta frequency range. The more similar brain activity in 129 AS mice, particularly in hippocampal EEG during the dark cycle may be reflective of the different behavioral and seizure susceptibility AS phenotypes.

In addition, there were strain specific differences in specific EEG frequencies. In line with visual inspection of the EEG activity in AS mice, we found significant increases, primarily in delta and theta power, in cortical and hippocampal EEG. Delta oscillations are characteristic of slow-wave sleep. Indeed, disruptions of sleep cycling are commonly reported in AS patients^26–28^ and also have been seen in *Ube3a* maternal deficient mice^29, 30^. Under physiological conditions the relative spectral power of theta waves is representative of the “on-line” state of the hippocampus or learning and memory^31, 32^. Studies from other neurological diseases such as schizophrenia and Alzheimer’s disease indicate a strong positive correlation between cognitive impairment and high theta power^33, 34^. The higher theta power that we observed in the cortical and hippocampal recordings from AS mice is seen in parallel with deficits in spatial learning and memory. An additional finding was decreased gamma power in the F1 AS hippocampal EEG. Gamma wave activity is involved in top-down attentional processing and perception of objects^35^, and may be linked to impaired behavior. To the best of our knowledge, a decrease in gamma power in AS mice has not previously been reported. Similar to our cortical EEG findings, enhanced delta power in V1 layer 4 recordings was recently described in the *Ube3a* ^*STOP/p*+^ and *Ube3a* ^*FLOX/p*+^ *::Gad2-Cre* mice compared to control mice^15^. Unlike our findings in cortical and hippocampal activity of the AS mice, no significant changes were found in the theta, alpha, beta, or gamma bands in this study^15^. Differences in spectral power analysis are likely due to differences in recording techniques and the amount of time/ time of day measured as well as potentially affected by differences in the genetic model, mouse background strain, and the frequencies analyzed.

Using spectral power analysis to quantify these changes in pilot studies from our lab, we have found that humans with AS have significantly increased delta and theta spectral power compared with age-matched controls (unpublished data). In line with these findings, high amplitude delta and theta EEG activity as assessed by qualitative visual inspection has been reported numerous times in individuals with AS^17–19, 36^. The appearance of abnormal EEG patterns has been found at early ages in individuals with AS^17^. We propose that recapitulation of such a predominant clinical feature in an AS model is an important biomarker for evaluating candidate therapeutics for AS. This will be particularly relevant if future studies reveal that normalization of these EEG rhythms correlates to cognitive improvement in models of AS.

In summary, we found strain-specific differences in the behavioral, EEG activity, and seizure phenotypes in the *Ube3a* maternal deletion mouse model. The B6 AS mice showed fairly robust differences in a number of behavioral tests and EEG studies, which will be valuable for screening the effects of novel therapeutics. The 129 AS mice demonstrated limited behavioral abnormalities but may be useful for screening novel therapeutics for seizure protection. While previous publications have found a hybrid breeding scheme can exacerbate phenotypes such as seizure severity^37^, there was no exacerbation of phenotypes in the F1 AS mice in our studies. While the F1 hybrid AS mice showed behavioral impairments and EEG polyspike activity, these differences compared to Wt mice were not as robust as with the B6 background. Together, our findings lay a strong groundwork for further studies to evaluate novel therapeutics for AS using the *Ube3a* mouse model.

## Materials and Methods

### Animals

*Ube3a* maternal deletion mice (AS model) were generated by Jiang and colleagues^9^. We maintained colonies of *Ube3a* maternal deficient (AS) mice on three different backgrounds: B6, 129 and F1 hybrid cross between B6 and 129. Breeding pairs of AS mice were purchased from Jackson Laboratory (Stock no. 016590 and Stock no. 004477) and maintained with mice generated from these two stocks. The B6 AS line was backcrossed to B6 for at least 8 generations for the establishment of the Jackson Laboratory colony, and the 129 line was generated in 129S7Sv/Ev stem cells and maintained on an unspecified 129 background. As we used the mice sent out from the Jackson Laboratory colony, they will be referred to as a general 129 background and not by a specific substrain. F1 hybrid mice were generated in our lab by crossing 129 AS females with B6 Wt males. Experimental mice were generated using paternal deficiency female mice as breeders. In all experiments, we used gender-balanced adult *Ube3a* maternal deficient and Wt littermates. Mice used for behavioral and seizure induction studies were group housed and tested during the light cycle. Mice recorded for EEG activity were single housed and recorded continuously for 5–7 days. Animal care and use were in accordance with regulations from the National Institute of Health Guidelines for the Care and Use of Laboratory animals and approved by the Institutional Animal Care and Use Committee at Baylor College of Medicine.

### Behavioral battery

3–7 months old male and female mice were tested in a behavioral battery as described below (Fig. 1) (n = 15–16/group). A battery of behavioral tests was performed in parallel for mice on the three different strains by two experimenters following the guidelines of Behavioral Core facilities at Baylor College of Medicine. Prior to each test, mice were acclimatized to the testing room for at least 30 minutes. Mice were given at least one hour rest in their home cages between tests completed on the same day.

#### Open field assay (OFA)

The OFA task evaluates locomotor activity, exploratory behavior, and anxiety level in rodents^38^. The OFA apparatus is a clear Plexiglass box measured 41 cm × 41 cm × 30.5 cm (Omnitech Electronics, Columbus, OH). During testing, white noise played at ~62 dB, and the lighting was dimmed to ~30 lux. Mice were placed in the center of the OFA box and allowed to explore for 30 minutes. The infrared sensors around the apparatus detect movements based on beams broken by the mouse to determine the location of the animal and can track horizontal and vertical movement, which were quantified using the Fusion software (Omnitech Electronics, Columbus, OH). A 25.6 cm × 25.6 cm area in the middle of the apparatus is defined as the center area. Total activity, total distance travelled and percent distance travelled in the center area were analyzed.

#### Elevated plus maze (EPM)

The EPM is used to screen for anxiety-like behavior and is a plus-shaped apparatus with two open and two closed arms (58 cm × 58 cm × 16 cm height of closed arms) that is elevated 40.5 cm from the ground in a testing room with ~60 dB white noise sound and 700–750 lux illumination. Mice were placed in the center zone facing one of the open arms and allowed 10 minutes for exploration. All movements were recorded and tracked by ANY-maze software. Time spent in the closed arms and total distance traveled were analyzed.

#### Marble burying

Marble burying measures repetitive behavior^16^. A clean cage is filled with approximately 20 cm of bedding and a rectangular pattern of 20 marbles are evenly spaced on the surface of the bedding. Mice were allowed 30 minutes to explore/bury the marbles. A marble was counted if more than 50% of the marble was buried.

#### Inverted screen

This test evaluates the muscle strength of all four limbs^39^. Briefly, mice were placed in the center of a metal grid with 1.2 cm square holes measuring 25.4 cm × 40.5 cm total. Mice are placed on the grid, which is gently shaken to alert the mouse to hold onto the metal grid and then slowly inverted so that the mouse is upside down. The screen was held steadily for 60s maximum over a padded surface. The latency to fall, or the maximum score of 60s if no fall occurred, was recorded for each mouse.

#### Wire hang

The wire hang test evaluates muscle strength as well as motor coordination^40^. The set up includes a wire (0.2 cm in diameter × 54.5 cm in length) stretched horizontally between two poles and elevated from a padded surface. The test starts when the mouse hangs onto the wire from its two forelimbs and lasts for 120s maximum. The latency to fall, or the maximum score of 120s if no fall occurred, was recorded for each animal.

#### Accelerating rotarod

The rotarod test evaluates motor learning skills and motor coordination in rodents^41^. Mice were trained for 4 trials per day for two consecutive days with a 30 min inter-trial interval. In each trial, mice were put on an accelerating rotarod (Ugo Basile, Varese, Italy) that increases in speed from 4–40 rpm over 5 minutes. Latency to fall, or time to the second full turn riding around the wheel, was recorded.

#### Novel object recognition (NOR)

The novel object recognition task tests learning and memory with a protocol over three days, modified from Antunes and Biala, 2012^42^. On days 1 and 2, mice were habituated for five minutes in a clean, empty testing arena (42 cm × 21.5 cm × 20 cm) followed by five minutes exploring two identical objects. On day 3, mice were habituated for five minutes in the empty testing arena and then tested with both the familiar object and a novel object for five minutes. Time spent exploring/interacting (e.g. direct contact or sniff with nose oriented towards object and within < 1 cm from the object) with each object was recorded by a blinded observer and the percent time interacting with each object was analyzed.

#### Fear conditioning (FC)

The FC test is used widely to assess associative learning and memory with contextual (hippocampal and amygdala-dependent) and cue (amygdala-dependent) tests^43^. For training, each mouse was placed in a sound attenuating chamber with a metal grid floor (Coulbourn Instruments, White Hall, PA) and given two pairings of tone (30 sec white noise at 85 ± 2 dB) and shock (0.72 mA for 2 sec) with 2 min for habituation and 120s intersound interval. 24 hours after training, mice were observed for 5 mins in the same chamber with no sound stimulus (contextual test). Two hours after the contextual test, mice were tested for cue fear memory with 3 minutes of no sound and 3 minutes of the tone in the chamber altered to have a plastic floor covering the metal grid, changed chamber shape with plastic inserts, vanilla odor, and red light in the testing room. For each test, the mouse was recorded and percent time freezing analyzed using the Coulbourn/Actimetrics FreezeFrame3 system (Coulbourn Instruments, White Hall, PA).

### EEG electrode implantation

Wt and AS mice were implanted for EEG recordings using three cortical screw electrodes and one hippocampal depth electrode (Plastics One, Roanoke, VA). Mice were implanted at 2 months of age (n = 3–7/group) as described previously^44, 45^. After anesthesia with isoflurane and positioning the mice in a stereotaxic frame, a 1–2 cm midline sagittal incision was made. The cortical recording electrodes (stereotaxic coordinates relative to bregma: −0.1 mm, −1.8 mm; −0.1 mm, 1.8 mm; −2.3mm, −1.8mm) and reference electrode anterior to the bregma were implanted subdurally through small holes drilled in the skull. The hippocampal electrode was positioned (−1.6 mm, 1.8 mm) at the depth of 1.8 mm. These electrodes were held in place with Metabond and dental cement. The ground electrode was sutured in the cervical paraspinous area. All electrodes were inserted into a 6 channel pedestal and connected to the commutator for recording. Mice were provided with slow release buprenorphine for pain relief and allowed to recover from surgery and the effects of analgesia for at least 1 week before long-term recording.

### EEG acquisition and analysis

Video synchronized EEG (vEEG) was recorded and analyzed using the Nicolet system (Natus, Pleasanton, CA) and Labchart V8 software (AD Instruments, Colorado Springs, CO), respectively. EEG data between 0.5 and 200 Hz were acquired at a sampling rate of 500 Hz. After 30 minutes acclimatization to the recording chamber, mice were recorded for 24 hrs/day for three to seven full days. We manually evaluated and quantified epileptiform activity as the number of polyspikes per day in Wt and AS mice. Spikes were defined as waveforms < 200 msec in duration and with a peak amplitude ≥ 2x baseline background amplitude, while a polyspike was defined as spikes crossing the baseline > 2 times.

### EEG spectral analysis

In order to quantify the difference between Wt and AS mice in baseline EEG activity, we used a Fast Fourier transform (FFT) algorithm to convert electrical activity to frequency with a FFT size of 512 and the Hann-Cosine method^46^. After visually inspecting the video EEG tracings and excluding artifacts from the data, we used Labchart V8 software for spectral analysis of EEG samples in Wt and AS mice (B6: n = 9–11 / group, 129: n = 8–10 / group; F1: n = 3–6 / group). Power analysis was performed on EEG baseline recordings for the full hour of recording between 12 pm–1 pm as a representation of activity during the light cycle and 12 am–1 am as a representation of activity during the dark cycle. For each animal analyzed, data from the same hour of the day over multiple days (1–3 days per animal) was averaged. We calculated the total power with frequencies from 0–50 Hz and also analyzed the spectral power of delta δ (0–4 Hz), theta θ (5–8 Hz), alpha α (8–12 Hz), beta β (14–29 Hz), and gamma γ (30–50 Hz) frequency bands.

### Audiogenic seizure (AGS) induction

We tested the AGS susceptibility of Wt and AS mice from all backgrounds (n = 11–12/group, 3–7 months old) using a 140 dB emitting alarm (SKU 49–728, RadioShack, Fort Worth, TX, USA). Briefly, mice were put in a sound-attenuating chamber with manual control of the noise and habituated for five minutes and then observed during the test with 1 minute sound off and 2 minutes sound on. A blinded observer recorded the appearance of and time to latency for wild running and tonic-clonic seizures as well as mortality.

### Kainate induction

For comparison of seizure threshold in the Wt and AS mice, we first completed a dose response curve with i.p. kainate on 7 week old Wt mice from each strain with doses from 15–50 mg/kg (n = 2–7/dose) for a one hour observation period to determine the optimal dose for each strain to evoke behavioral seizures as there is variability in mouse strain sensitivity to chemical-induced convulsions. Kainate (Tocris Bioscience, Minneapolis, MN) was used to induce behavioral seizures at the following concentrations in both Wt and AS mice (n = 11–13 mice/group): B6 (25 mg/kg), 129 (40 mg/kg), F1 (35 mg/kg). Following kainate injection, seizures were scored by blinded observers and mice were monitored for an hour. Seizure stage was determined based on a modified Racine scale^47^, stage 1: rigid posture or immobility, mouth moving; stage 2: tail clonus; stage 3: partial body clonus with forelimb or hindlimb clonus, head bobbing; stage 4: rearing; stage 4.5: severe whole body clonus while retaining posture; stage 5 seizure: rearing and falling; and stage 6: loss of posture, wild-running, and jumping.

### Statistics

All statistical analyses were done using GraphPad Prism 6.0. Student’s t-test was used to compare between genotypes within the strain background. Student’s t-test was performed with Welch’s correction where variances were significantly different. Rotarod results were analyzed with 2-way ANOVA and Bonferroni posttest. EEG spectral analysis results were analyzed with 2-way ANOVA and Sidak’s multiple comparisons for posthoc tests to determine differences within frequencies. Fisher’s exact test was used in seizure percentage analysis. Graphs display group mean with errors bars ± SEM; *p < 0.05; **p < 0.01; ***p < 0.001.

### Data availability

The datasets generated during and/or analysed during the current study are available from the corresponding author on reasonable request.

## Electronic supplementary material

Supplemental Figures
