# Supplementary material for: Strain-dependence of the Angelman Syndrome phenotypes in Ube3a maternal deficiency mice
Source: Sci Rep. 2017 Aug 16;7:8451. doi: 10.1038/s41598-017-08825-x (PMC5559514; doi:10.1038/s41598-017-08825-x)
Supplement: Supplementary file 1 — Supplemental Figures [file 41598_2017_8825_MOESM1_ESM.pdf]

Strain-dependence of the Angelman Syndrome phenotypes in *Ube3a* maternal deficiency mice

Heather A. Born[1], An T. Dao[1], Amber T. Levine[1,2], Wai Ling Lee[1], Natasha M. Mehta[1,3], Shubhangi Mehra[1,3], Edwin J. Weeber[4], Anne E. Anderson*[1,2,5]

[1]Cain Foundation Laboratories, Jan and Dan Duncan Neurological Research Institute at Texas Children's Hospital and Department of Pediatrics, Baylor College of Medicine, Houston, TX, USA
[2]Department of Neuroscience, Baylor College of Medicine, Houston, TX, USA
[3]Rice University, Houston, TX, USA
[4]USF Health Byrd Alzheimer's Institute, Department of Molecular Pharmacology and Physiology, University of South Florida, Tampa, FL, USA
[5]Department of Neurology, Baylor College of Medicine, Houston, TX, USA
* Correspondence to annea@bcm.edu

Supplemental Data:

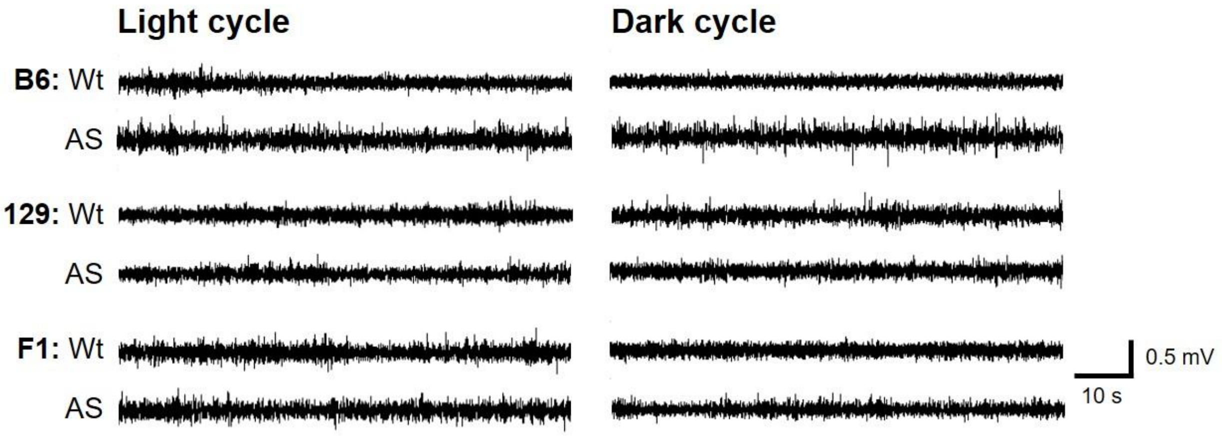

Figure S1. Representative cortical EEG traces from Wt and AS mice on B6, 129, and F1 backgrounds during the light cycle and dark cycle time points used for spectral analysis.

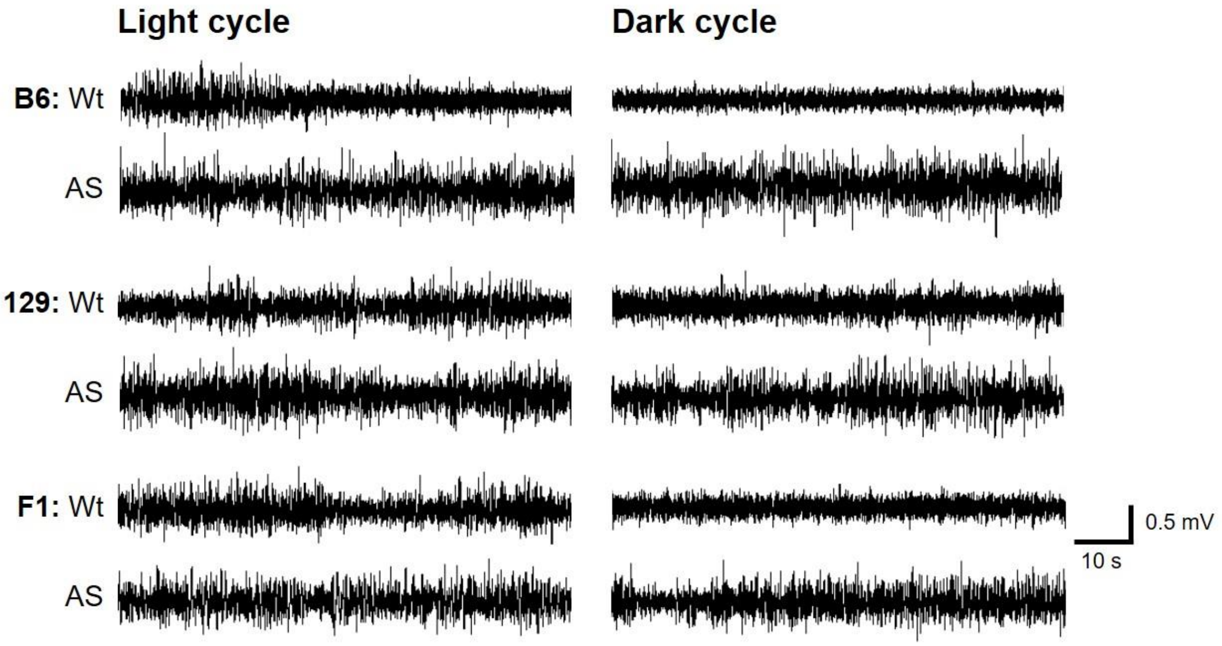

Figure S2. Representative hippocampal EEG traces from Wt and AS mice on B6, 129, and F1 backgrounds during the light cycle and dark cycle time points used for spectral analysis.

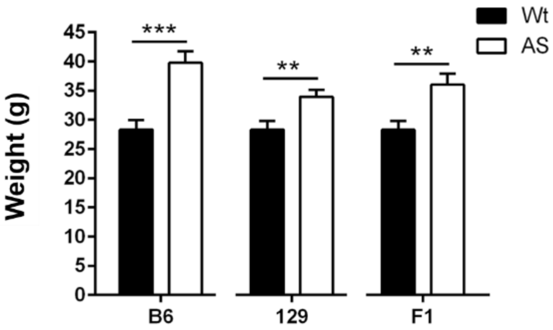

Figure S3. Weight was measured in mice used for behavioral assessment at 3-7 months of age in gender-balanced groups of Wt and AS mice from B6, 129, and F1 hybrid backgrounds. AS mice weighed significantly more than age-matched Wt mice, regardless of background (B6, Wt: 28.30 ± 1.694 g, AS: 39.78 ± 1.985 g, $p = 0.001$; 129, Wt: 28.34 ± 1.450 g, AS: 33.97 ± 1.168 g, $p = 0.0053$; F1, Wt: 28.30 ± 1.516 g, AS: 36.01 ± 1.929 g, $p = 0.0039$). Student's t-test was used to compare between genotypes and data are presented as the mean ± SEM; n=15-16 mice/group; **p<0.01, ***p < 0.001.
